# Effectiveness of community case management of malaria on severe malaria and inpatient malaria deaths in Zambia: a dose–response study using routine health information system data

**DOI:** 10.1186/s12936-023-04525-2

**Published:** 2023-03-17

**Authors:** Ruth A. Ashton, Busiku Hamainza, Chris Lungu, Marie-Reine I. Rutagwera, Travis Porter, Adam Bennett, Michael Hainsworth, Sarah Burnett, Kafula Silumbe, Hannah Slater, Thomas P. Eisele, John M. Miller

**Affiliations:** 1 Center for Applied Malaria Research and Evaluation, Tulane University School of Public Health and Tropical Medicine, 1440 Canal Street, Suite 2300, New Orleans, LA USA; 2 National Malaria Elimination Centre, Zambia Ministry of Health, Lusaka, Zambia; 3 PATH Malaria Control and Elimination Partnership in Africa (MACEPA), Lusaka, Zambia; 4 PATH MACEPA, Seattle, WA USA

**Keywords:** Community case management, Severe malaria, Impact evaluation, Routine health information system, Community health workers, Zambia

## Abstract

**Background:**

Community case management of malaria (CCM) has been expanded in many settings, but there are limited data describing the impact of these services in routine implementation settings or at large scale. Zambia has intensively expanded CCM since 2013, whereby trained volunteer community health workers (CHW) use rapid diagnostic tests and artemether-lumefantrine to diagnose and treat uncomplicated malaria.

**Methods:**

This retrospective, observational study explored associations between changing malaria service point (health facility or CHW) density per 1000 people and severe malaria admissions or malaria inpatient deaths by district and month in a dose–response approach, using existing routine and programmatic data. Negative binomial generalized linear mixed-effect models were used to assess the impact of increasing one additional malaria service point per 1000 population, and of achieving Zambia’s interim target of 1 service point per 750 population. Access to insecticide-treated nets, indoor-residual spraying, and rainfall anomaly were included in models to reduce potential confounding.

**Results:**

The study captured 310,855 malaria admissions and 7158 inpatient malaria deaths over 83 districts (seven provinces) from January 2015 to May 2020. Total CHWs increased from 43 to 4503 during the study period, while health facilities increased from 1263 to 1765. After accounting for covariates, an increase of one malaria service point per 1000 was associated with a 19% reduction in severe malaria admissions among children under five (incidence rate ratio [IRR] 0.81, 95% confidence interval [CI] 0.75–0.87, p < 0.001) and 23% reduction in malaria deaths among under-fives (IRR 0.77, 95% CI 0.66–0.91). After categorizing the exposure of population per malaria service point, there was evidence for an effect on malaria admissions and inpatient malaria deaths among children under five only when reaching the target of one malaria service point per 750 population.

**Conclusions:**

CCM is an effective strategy for preventing severe malaria and deaths in areas such as Zambia where malaria diagnosis and treatment access remains challenging. These results support the continued investment in CCM scale-up in similar settings, to improve access to malaria diagnosis and treatment.

**Supplementary Information:**

The online version contains supplementary material available at 10.1186/s12936-023-04525-2.

## Background

An estimated 229 million malaria cases occurred worldwide in 2019, leading to 409,000 malaria-attributable deaths [1], nearly all of which could have been prevented through prompt diagnosis and treatment. Ensuring universal access to malaria diagnostic and treatment services for populations at risk of malaria is a core component of the Global Malaria Technical Strategy [2], Establishing community-level malaria diagnostic and treatment services is recommended as an important step in improving access to effective malaria case management, particularly in rural areas [3]. Community-level health service delivery has seen expansion using variety of models; from disease-specific initiatives relying on volunteer community members, to approaches where community health workers (CHWs) provide an integrated package of services and are formal, salaried members of health staff [4, 5]. While commitment to continued expansions of community-based health services remains strong, there are limited data describing the impact of these community-based services in routine implementation settings at large-scale [6], particularly in describing the impact on malaria morbidity and mortality [7].

Severe malaria results from progression of uncomplicated malaria, and may manifest as severe malarial anaemia, cerebral malaria, or respiratory distress syndrome, or a combination of these [8]. All cases of severe malaria should be preventable in the presence of prompt and effective diagnosis and treatment for uncomplicated malaria. An individual-level meta-analysis estimated that almost half of severe malarial anaemia cases could be prevented if patients seek diagnosis and treatment within 24 h of illness onset [9], emphasizing the importance of timely treatment in preventing severe disease and death. The same analysis also highlighted the association between increasing travel time to health facilities and increased odds of severe malaria [9], a challenge which could be addressed by expanding CHW networks in rural settings. The lack of evidence evaluating the impact of community-level interventions on malaria morbidity and mortality was highlighted by a 2013 Cochrane review [10], but more recently supplemented by a review reporting that community-delivered malaria interventions were found to be associated with reduced malaria death rates and reduced parasite prevalence [11]. However, the evidence base for impact of community-based case management on malaria remains focused primarily on high-risk groups (children under five years, pregnant women) in sub-Saharan Africa, with most studies focused on relatively-small scale randomized trials or evaluations. Heterogeneity in community-delivery models are a further challenge in the generalizability of existing evidence to other settings, particularly where interventions are applied in controlled trial settings which may not be feasible in routine implementation contexts.

Zambia first piloted community case management for malaria (CCM) in 2009 in selected districts in Lusaka and Southern Province [12]. CCM has been intensively expanded across multiple provinces of Zambia since 2013, with coordinated support from a range of partners to the Ministry of Health’s National Malaria Elimination Centre (NMEC). Zambia’s current Malaria Elimination Strategic Plan states that all suspected malaria cases should receive parasitological testing, and all confirmed cases should receive effective anti-malarial treatment within 24 h with artemisinin-based combination therapy [13]. To accomplish this, the National Community Health Strategy has the goal of providing all Zambians with quality basic health services within 5 km or one hour’s travel of their home by 2021 [14]. The NMEC initially worked towards a target in pre-elimination areas of one CHW providing malaria diagnosis and treatment per 750 people, but this may be revised to one CHW per 500 people in higher burden settings. The proportion of febrile children under five receiving anti-malarials from CHWs (among all those receiving anti-malarials) has increased from 2.1% in 2010 to 22% by 2018 [15].

It is hypothesized that increasing the number of malaria diagnosis and treatment service points per population through CHW expansion reduces delays in treatment-seeking, reducing the proportion of individuals who progress from uncomplicated to severe malaria or malaria-related death. The aim of this analysis was to evaluate the impact of CCM expansion on severe malaria admissions and inpatient malaria deaths during the period 2015 to 2020, using existing routine surveillance data and programme records. Furthermore, this analysis sought to assess if reaching the target of one malaria service point (CHW or health facility) per 750 population was associated with measured reductions in severe malaria morbidity.

## Methods

### Community case management of malaria in Zambia

CCM is conducted in Zambia by trained volunteer CHWs that operate under the supervision of a health facility that supplies them with rapid diagnostic tests (RDTs) and the first-line antimalarial artemether-lumefantrine to treat uncomplicated malaria episodes in their community. Individuals with symptoms of severe malaria are referred by CHWs to the nearest health facility. CHWs providing CCM are part of the public health system in Zambia and report monthly malaria surveillance data to the NMEC Malaria Rapid Reporting System (MRRS).

### Study design

This study was designed to evaluate of the effectiveness of changes in malaria service point density per population, as a proxy for scale up of community-case management services, on confirmed malaria inpatient admissions and deaths among inpatients with confirmed malaria. Analysis focused on seven of Zambia’s ten provinces, which experienced any roll-out of CCM during January 2015 to May 2020. Central, Lusaka and Southern provinces were excluded due to either limited CCM introduction during the study period (Central and Lusaka), or extensive CCM scale up prior to 2015 (Southern). The study was retrospective and observational, using a dose–response approach to investigate associations between a continuous measure of malaria service point density over time and malaria admissions and mortality by district and month.

### Primary exposure variables

The primary exposure was defined as the number of malaria service points per 1000 population for a district-month. This indicator considers either a health facility or a CHW as a malaria service point, irrespective of staffing or ‘level’ within the health system.

Data from the NMEC’s MRRS were used to calculate the number of community-level service points operating in a district each month. The MRRS includes malaria cases identified through both active and passive surveillance methods by each CHW each month, as well as information on their first and most recent report. Each CHW is associated with a ‘parent’ health facility in the MRRS, the supervising and usually geographically closest facility, allowing matching of each CHW to a district. CHWs were removed from monthly district service point counts for any periods of inactivity, defined as greater than six months when either: (1) no malaria tests were completed (through active and passive surveillance); or (2) malaria testing data was missing. The number of health facilities operating per month were extracted from the Zambian Ministry of Health Management Information System (HMIS), then processed in the same manner as the CHW-level data to exclude inactive site. The sum of the active facilities and active CHWs was calculated for each district and month to give the total malaria service points, then divided by a temporally static district population estimate (see below) to give the exposure indicator of malaria service points per 1000 population.

The GRID3 modelled 2019 population raster surface at 100 m resolution was used to estimate total population by district [16, 17]. The modelled total Zambian population from GRID3 was rescaled to 18 million, in accordance with the official Zambian population estimate for 2020 (zamstats.gov.zm).

### Primary outcome variables

Malaria morbidity outcome data were extracted from the HMIS, which collates monthly clinical data by health facility. Primary outcomes for this analysis were: (1) Under-five (U5) inpatient admissions with severe confirmed malaria (hereafter termed U5 severe malaria admissions); (2) all-ages inpatient admissions with severe confirmed malaria (hereafter termed all-age severe malaria admissions); (3) U5 deaths among inpatient admissions with confirmed malaria (hereafter termed U5 malaria deaths); (4) all-ages deaths among inpatient admissions with confirmed malaria (hereafter termed all-age malaria deaths). Data were extracted in August 2020 for the period January 2015 to May 2020, inclusive.

HMIS data cleaning involved identification and replacement of anomalous values, but used a ‘light touch’ approach aiming to retain as much data as possible and minimize any modifications. Briefly, timeseries plots of the key outcome indicators were prepared for each health facility and apparent anomalies identified by visual inspection. Raw data were inspected to assess feasibility of the anomalous value, comparing against outpatient confirmed malaria case counts and all-cause inpatient and outpatient totals. If determined to be anomalous (0.05% of all facility-month observations), the value was replaced by the mean value for the facility in the same year and transmission season (December-May or June-November). All data for one district in Eastern Province were removed due to multiple irreconcilable errors. Facility-month totals for each outcome indicator were aggregated to district-month.

### Potential confounding variables

The total number of facilities in a district-month providing inpatient services was estimated from HMIS data, where any facility reporting inpatient utilization of at least one patient in the calendar year facility was determined to offer inpatient services.

Insecticide treated net (ITNs) and indoor-residual spraying (IRS) indicators were included in models to account for access to other prevention interventions. Vector control data were compiled from existing programmatic data sources (NMEC, MACEPA and the PMI VectorLink Project) and from HMIS. Briefly, ITNs distributed through continuous and mass distribution channels were totaled by district and month. A rolling total ITNs distributed in the prior 12 months by district was calculated to reflect availability of nets within communities. This simple approach avoided the need to use more complex assumptions around durability, insecticide decay, and usage over time of ITNs. This resulted in a covariate of ITN availability over space and time, which was divided by district population to estimate ITNs distributed per person in the previous 12 months. IRS campaign dates, number of sprayed structures and population protected were available at facility-catchment or district level. Data were summarized to district-month level, calculating the proportion of the district population receiving IRS in the campaign. The proportion of the population protected by IRS was maintained for 6 months after the campaign end date, then dropped to 0% protected [18]. Pirimiphos methyl was used in all IRS-receiving districts from 2014 to 2018, while clothianidin and DDT were used in 2019. Other malaria interventions that may have been relevant were either targeted to provinces not included in this analysis (mass drug administration in Southern province) or began implementation after the study period (pre-referral rectal artesunate).

Ten new districts were established during the study period. Data for the whole study period were allocated according to the new district structure. Vector control data were reallocated according to proportion of the parent district’s population in each of the new districts, assuming ITNs and IRS were delivered evenly across the parent district.

Monthly total rainfall data was downloaded from CHIRPs v2.0 at ~ 5 km resolution [19], then summarized to district-level by calculating mean of all district pixels. Monthly rainfall anomaly was defined as the difference between the monthly district rainfall and mean rainfall for the specific district and calendar month, then standardized to mean 0 and standard deviation of 1 to facilitate model fitting. Biologically plausible lags (1–3 months) were calculated for standardized rainfall anomalies.

### Analytical approach

A dose–response relationship was hypothesized between the number of malaria diagnosis and treatment service points (facilities or CHWs, the ‘dose’) and the number of inpatient malaria admissions or deaths among malaria admissions (the ‘response’) by district and month. Dose–response models were explored using both a continuous exposure, which allows interpretation of the impact of adding one additional malaria service points unit per 1000, and a categorized exposure variable to compare districts approaching and achieving the NMEC interim target of at least 1 service point per 750 population.

A negative binomial generalized linear mixed-effects model with a log link function was developed for each of the four outcomes. All models included a district random effect to account for repeated measures, and a logged district population offset to adjust for differences in district populations.

To account for changes in vector control interventions by district and over time, ITN and IRS indicators were included in models a priori. Models also included a priori one rainfall-derived indicator (lags of 1–3 months’ rainfall anomalies) to account for climate-driven changes in transmission over the study period, and the number of facilities with inpatient services. A one-month lag of the primary outcome was included to account for temporal autocorrelation. Additional fixed effects (calendar month, year, province) were tested with best fit ascertained according to the Akaike Information Criterion. The final covariate set for each of the four continuous outcomes was also used in models with categorized exposure variable. Analysis was completed in R version 4.0.3, with models developed using the lme4 package. Maps were prepared in QGIS version 2.12.

## Results

**Fig. 1:**
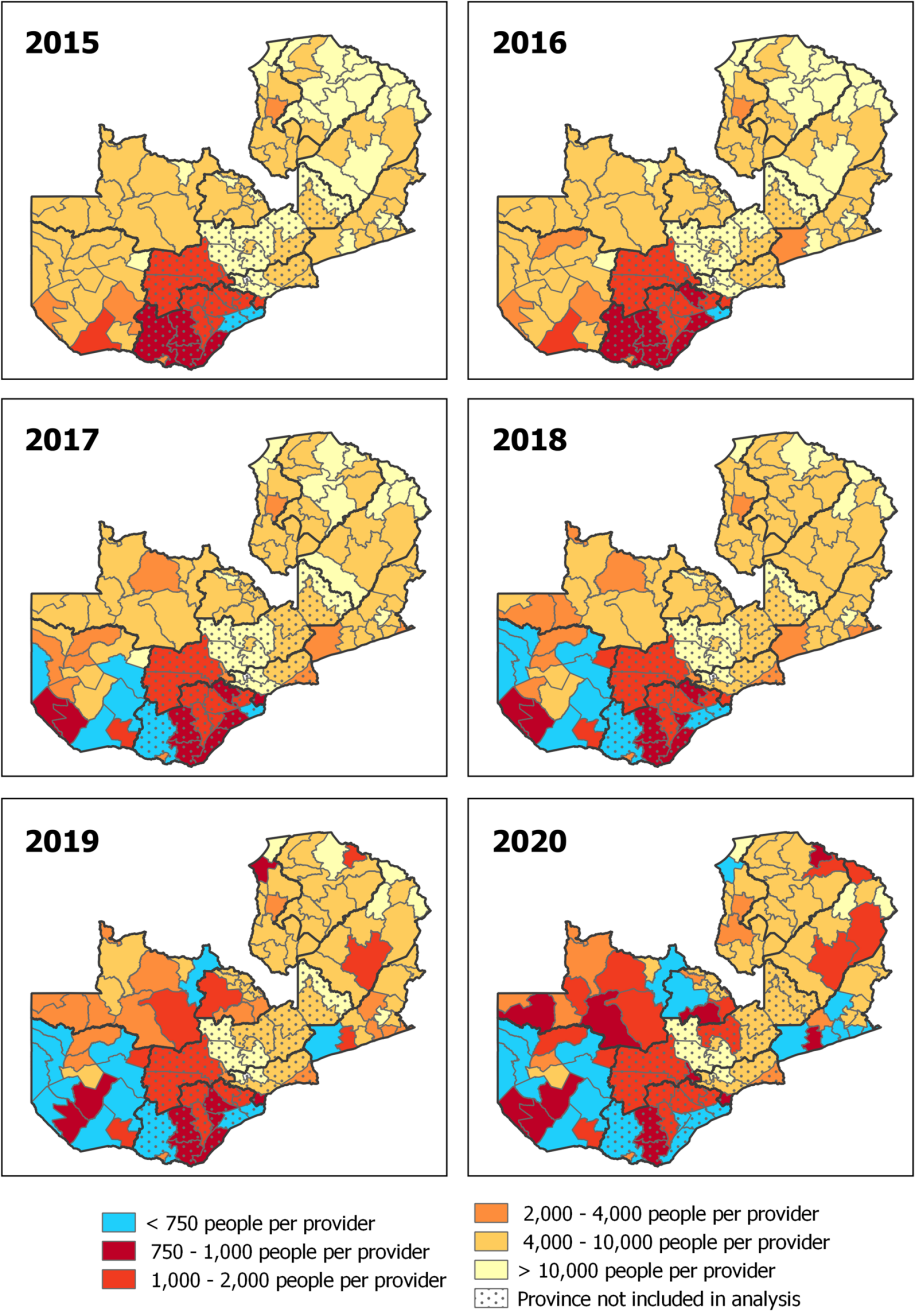
Total population per malaria service point (either health facility or community health worker), by district. Blue shading indicates a district reaching the target of 1 malaria service point per 750 population. Data are presented for January of each year from 2015 to 2020

Data from 83 districts (seven provinces) and 65 months were used for this dose response analysis, covering an area with a population of 10.3 million. Descriptive summaries of key variables (exposure, outcomes, vector control, rainfall) by year are available in Table 1. Change in population per malaria service point by district is shown in Fig. 1. The observed increase in malaria service points were primarily due to expansion of CHW services (from 43 to 4503), complemented by concurrent moderate increase in health facility numbers (1263 to 1765) during the study period (Additional file 1: Fig. S1).

**Table 1:** Summary of key variables over the study period, January 2015 to May 2020*

	Year					
	2015	2016	2017	2018	2019	2020^a^
Exposure						
Number CHWs operating	43	436	802	1712	4243	4503
Number Health facilities operating	1263	1407	1547	1616	1714	1765
Total malaria service points (facility or CHW)	1306	1843	2349	3328	5957	6268
% districts with ≥ 1 malaria service point per 750 people	0%	3.6%	7.2%	8.4%	19.3%	20.5%
Number (annual %) district-months with:						
> 2000 people per malaria service point	984 (98.8%)	958 (96.2%)	901 (90.5%)	839 (84.2%)	667 (67.0%)	241 (58.1%)
1001–2000 people per malaria service point	12 (1.2%)	18 (1.8%)	14 (1.4%)	33 (3.3%)	98 (9.8%)	53 (12.8%)
751–1000 people per malaria service point	0 (0.0%)	7 (0.7%)	27 (2.7%)	35 (3.5%)	62 (6.2%)	34 (8.2%)
< 750 people per malaria service point	0 (0.0%)	13 (1.3%)	54 (5.4%)	89 (8.9%)	169 (17.0%)	87 (21.0%)
Outcomes						
Total malaria admissions, all ages	71,184	60,175	50,331	46,725	49,464	32,976
Total malaria admissions, U5s	36,742	28,978	25,106	22,391	24,695	15,437
Total deaths among malaria admissions, all-ages	1,734	1,341	1,091	1,017	1,166	809
Total deaths among malaria admissions, U5s	991	717	591	521	611	441
Covariates						
Total ITNs distributed in year	847,941	505,198	4,700,685	2,480,013	652,520	191,262
% population received IRS at any time in calendar year	41.4%	45.1%	52.5%	53.8%	72.2%	13.6%
Total rainfall in mm	81,621	83,243	105,635	87,654	80,845	63,274
Facilities with inpatient services	943	1132	1293	1296	1371	1455

^*^Number of malaria service points, CHWs, and facilities reflects the status in December each year, or in May 2020. Total malaria admissions and deaths reflect the totals across all 83 study districts for the whole calendar year, or January to May (peak transmission season) for 2020. Note that since IRS is usually completed during October-January, the proportion of population receiving IRS during 2020 appears artificially low due to restriction of 2020 data to January-May only

^a^2020 data for January to May only, corresponds with peak transmission season

A total of 310,855 malaria admissions and 7158 deaths among admissions with confirmed malaria occurred in the seven study provinces (Additional file 1: Fig. S1). Over the whole study period, 412 district-months had ≤ 750 people per malaria service point, 165 district-months had 751–1,000 people per service point, 228 district-months 1001–2000 people per service point, and 4590 district-months > 2000 people per service point.

Final dose–response regression models for inpatient admission outcomes (among U5s and among all ages) included categorical variables for month, year and province, as well as a priori variables to account for vector control activities (total ITNs distributed in the previous 12 months and proportion of the district population protected by IRS) and rainfall anomalies. After accounting for these covariates, it was estimated that an increase of one malaria service point per 1000 population was associated with a 19% reduction in U5 severe malaria admissions (incidence rate ration [IRR] 0.81, 95% confidence interval [CI] 0.75–0.87, p < 0.001), and a 16% reduction in all-age severe malaria admissions (IRR 0.84, 95% CI 0.78–0.90, p < 0.001) (Table 2).

**Table 2:** Dose–response negative binomial generalized linear mixed-effects models describing association between the number of malaria service points per 1000 population as a continuous variable and the four primary outcome indicators

	Malaria admissions, children < 5 years			Malaria admissions, all ages			Inpatient malaria deaths, children < 5 years			Inpatient malaria deaths, all ages		
	IRR	95% CI	p	IRR	95% CI	p	IRR	95% CI	p	IRR	95% CI	p
Malaria service points per 1000 population	0.81	0.75, 0.87	< 0.001	0.84	0.78, 0.90	< 0.001	0.77	0.66, 0.91	0.002	0.78	0.68, 0.89	< 0.001
One-month lag of outcome variable	1.01	1.01, 1.01	< 0.001	1.01	1.00, 1.01	< 0.001	1.10	1.07, 1.13	< 0.001	1.09	1.07, 1.11	< 0.001
Total ITNs distributed in previous 12 months	0.84	0.77, 0.92	< 0.001	0.84	0.77, 0.91	< 0.001	0.69	0.58, 0.83	< 0.001	0.74	0.63, 0.86	< 0.001
% population protected by IRS	0.87	0.78, 0.97	0.014	0.85	0.76, 0.94	0.001	0.91	0.73, 1.14	0.431	0.96	0.80, 1.15	0.650
Rainfall anomaly^a^	1.03	1.00, 1.05	0.021	1.03	1.01, 1.06	0.003	0.97	0.92, 1.01	0.173	0.98	0.94, 1.02	0.356
Number of health facilities with inpatient services	1.00	0.99, 1.00	0.316	0.99	0.99, 1.00	0.060	0.99	0.98, 1.00	0.078	0.99	0.98, 1.01	0.333
Month (categorical)												
January	Ref.	–	–	Ref.	–	–	Ref.	–	–	Ref.	–	–
February	0.86	0.77, 0.95	0.003	0.82	0.75, 0.91	< 0.001	0.82	0.67, 1.00	0.052	0.82	0.69, 0.97	0.019
March	0.99	0.90, 1.09	0.846	0.98	0.89, 1.08	0.693	0.97	0.80, 1.19	0.794	0.94	0.80, 1.11	0.461
April	1.05	0.95, 1.17	0.312	1.01	0.92, 1.11	0.795	0.87	0.71, 1.06	0.162	0.93	0.78, 1.09	0.360
May	0.96	0.86, 1.06	0.384	0.93	0.84, 1.02	0.119	0.89	0.73, 1.08	0.231	0.91	0.77, 1.07	0.255
June	0.65	0.58, 0.73	< 0.001	0.60	0.54, 0.66	< 0.001	0.62	0.49, 0.79	< 0.001	0.66	0.55, 0.80	< 0.001
July	0.49	0.44, 0.55	< 0.001	0.45	0.40, 0.50	< 0.001	0.49	0.38, 0.63	< 0.001	0.54	0.44, 0.66	< 0.001
August	0.46	0.41, 0.52	< 0.001	0.40	0.36, 0.44	< 0.001	0.47	0.36, 0.61	< 0.001	0.49	0.40, 0.61	< 0.001
September	0.52	0.47, 0.59	< 0.001	0.46	0.41, 0.51	< 0.001	0.48	0.37, 0.62	< 0.001	0.51	0.42, 0.63	< 0.001
October	0.61	0.55, 0.68	< 0.001	0.57	0.51, 0.63	< 0.001	0.57	0.45, 0.71	< 0.001	0.56	0.47, 0.68	< 0.001
November	0.65	0.59, 0.73	< 0.001	0.63	0.56, 0.69	< 0.001	0.56	0.44, 0.70	< 0.001	0.55	0.45, 0.66	< 0.001
December	0.83	0.75, 0.93	0.001	0.80	0.72, 0.88	< 0.001	0.74	0.59, 0.93	0.008	0.72	0.60, 0.86	< 0.001
Year (categorical)												
2015	Ref.	–	–	Ref.	–	–	Ref.	–	–	Ref.	–	–
2016	0.84	0.78, 0.91	< 0.001	0.88	0.82, 0.95	0.001	0.72	0.61, 0.84	< 0.001	0.79	0.69, 0.90	< 0.001
2017	0.83	0.76, 0.90	< 0.001	0.87	0.81, 0.94	0.001	0.66	0.56, 0.79	< 0.001	0.69	0.60, 0.79	< 0.001
2018	0.79	0.73, 0.87	< 0.001	0.85	0.79, 0.92	< 0.001	0.66	0.56, 0.79	< 0.001	0.72	0.62, 0.83	< 0.001
2019	0.78	0.71, 0.86	< 0.001	0.80	0.74, 0.88	< 0.001	0.68	0.56, 0.82	< 0.001	0.73	0.62, 0.86	< 0.001
2020	0.94	0.82, 1.07	0.330	1.02	0.90, 1.15	0.803	0.96	0.74, 1.25	0.779	0.92	0.74, 1.14	0.431
Province												
Copperbelt	Ref.	–	–	Ref.	–	–	Ref.	–	–	Ref.	–	–
Eastern	2.29	0.99, 5.29	0.052	1.80	0.78, 4.14	0.167	2.35	0.99, 5.59	0.053	1.24	0.53, 2.91	0.621
Luapula	4.85	2.08, 11.3	< 0.001	3.91	1.68, 9.11	0.002	6.30	2.63, 15.1	< 0.001	3.28	1.39, 7.75	0.007
Muchinga	3.07	1.24, 7.63	0.016	2.51	1.01, 6.21	0.047	2.28	0.89, 5.83	0.085	1.29	0.51, 3.27	0.586
Northern	2.53	1.08, 5.91	0.032	2.10	0.90, 4.89	0.086	2.48	1.03, 5.92	0.042	1.50	0.64, 3.55	0.353
North Western	4.23	1.78, 10.1	0.001	3.82	1.61, 9.05	0.002	3.25	1.33, 7396	0.010	2.33	0.97, 5.61	0.060
Western	1.07	0.48, 2.39	0.868	1.20	0.54, 2.67	0.655	1.83	0.80, 4.23	0.154	1.34	0.59, 3.04	0.477

^a^Rainfall anomalies are lagged and standardized. 3-month lags were used in inpatient deaths models, and 2-month lag in admissions models

Models where malaria deaths were the outcome included the same covariates as the severe malaria admissions models, in addition to a covariate describing the number of inpatient facilities in the district-month. The model estimated that an increase of one malaria service point per 1000 population was associated with a 23% reduction in U5 malaria deaths (IRR 0.77, 95% CI 0.66–0.91, p = 0.002), and a 22% reduction in all-ages malaria deaths (IRR 0.78, 95% CI 0.68–0.89, p < 0.001) after accounting for potential confounding factors (Table 2).

For both malaria admission outcomes and U5 malaria deaths, models using a categorized variable to describe malaria service point density by district-month found that a significant association was observed only when malaria service point density exceeded the NMEC-defined target of at least one service point per 750 people (Table 3). The all-ages malaria deaths model shows a similar pattern, remained non-significant when exceeding one service point per 750 people.

**Table 3:** Dose–response negative binomial generalized linear mixed-effects models describing association between categories of malaria service point density and the four primary outcome indicators

	Malaria admissions, children < 5 years			Malaria admissions, all ages			Inpatient malaria deaths, children < 5 years			Inpatient malaria deaths, all ages		
	IRR	95% CI	p	IRR	95% CI	p	IRR	95% CI	p	IRR	95% CI	p
Population per malaria service point												
> 2000	Ref.	–	–	Ref.	–	–	Ref.	–	–	Ref.	–	–
2000–1000	1.04	0.90, 1.20	0.636	1.00	0.87, 1.14	0.954	1.08	0.79, 1.48	0.635	1.18	0.91, 1.52	0.203
1000–750	0.98	0.80, 1.21	0.875	0.98	0.81, 1.19	0.865	1.14	0.74, 1.76	0.552	1.00	0.70, 1.43	0.985
< 750	0.72	0.60, 0.85	< 0.001	0.74	0.63, 0.87	< 0.001	0.64	0.44, 0.95	0.025	0.79	0.58, 1.08	0.146
One-month lag of outcome variable	1.01	1.01, 1.01	< 0.001	1.01	1.00, 1.01	< 0.001	1.10	1.07, 1.13	< 0.001	1.09	1.07, 1.11	< 0.001
Total ITNs distributed in previous 12 months	0.84	0.77, 0.92	< 0.001	0.84	0.77, 0.91	< 0.001	0.70	0.58, 0.84	< 0.001	0.74	0.63, 0.86	< 0.001
% population protected by IRS	0.87	0.78, 0.97	0.012	0.84	0.76, 0.93	0.001	0.91	0.73, 1.15	0.432	0.95	0.79, 1.15	0.620
Rainfall anomaly^a^	1.03	1.00, 1.05	0.023	1.03	1.01, 1.06	0.003	0.97	0.92, 1.01	0.169	0.98	0.94, 1.02	0.365
Number of health facilities with inpatient services	1.00	0.99, 1.00	0.234	0.99	0.98, 1.00	0.038	0.99	0.98, 1.00	0.073	0.99	0.98, 1.01	0.285
Month (categorical)												
January	Ref.	–	–	Ref.	–	–	Ref.	–	–	Ref.	–	–
February	0.86	0.77, 0.95	0.003	0.82	0.75, 0.91	< 0.001	0.82	0.67, 1.00	0.056	0.82	0.69, 0.97	0.019
March	0.99	0.90, 1.09	0.840	0.98	0.89, 1.08	0.680	0.98	0.80, 1.19	0.809	0.94	0.80, 1.11	0.464
April	1.05	0.95, 1.16	0.342	1.01	0.92, 1.11	0.838	0.86	0.71, 1.06	0.154	0.92	0.78, 1.09	0.349
May	0.96	0.86, 1.06	0.377	0.93	0.84, 1.02	0.119	0.89	0.73, 1.08	0.230	0.91	0.77, 1.07	0.247
June	0.65	0.58, 0.73	< 0.001	0.59	0.53, 0.66	< 0.001	0.62	0.49, 0.79	< 0.001	0.66	0.55, 0.80	< 0.001
July	0.49	0.44, 0.55	< 0.001	0.45	0.40, 0.50	< 0.001	0.49	0.39, 0.63	< 0.001	0.54	0.44, 0.66	< 0.001
August	0.46	0.41, 0.52	< 0.001	0.40	0.35, 0.44	< 0.001	0.47	0.37, 0.61	< 0.001	0.49	0.40, 0.60	< 0.001
September	0.52	0.46, 0.59	< 0.001	0.46	0.41, 0.51	< 0.001	0.48	0.37, 0.62	< 0.001	0.51	0.41, 0.62	< 0.001
October	0.61	0.54, 0.68	< 0.001	0.56	0.51, 0.63	< 0.001	0.57	0.45, 0.72	< 0.001	0.56	0.47, 0.68	< 0.001
November	0.65	0.58, 0.72	< 0.001	0.62	0.56, 0.69	< 0.001	0.55	0.44, 0.70	< 0.001	0.55	0.45, 0.66	< 0.001
December	0.83	0.74, 0.92	0.001	0.79	0.71, 0.88	< 0.001	0.74	0.59, 0.92	0.007	0.72	0.59, 0.86	< 0.001
Year (categorical)												
2015	Ref.	–	–	Ref.	–	–	Ref.	–	–	Ref.	–	–
2016	0.84	0.78, 0.91	< 0.001	0.88	0.82, 0.95	0.001	0.72	0.61, 0.84	< 0.001	0.79	0.69, 0.90	< 0.001
2017	0.83	0.76, 0.90	< 0.001	0.87	0.80, 0.94	< 0.001	0.66	0.56, 0.79	< 0.001	0.69	0.60, 0.79	< 0.001
2018	0.79	0.72, 0.86	< 0.001	0.85	0.78, 0.92	< 0.001	0.66	0.55, 0.79	< 0.001	0.71	0.62, 0.83	< 0.001
2019	0.77	0.70, 0.84	< 0.001	0.79	0.72, 0.86	< 0.001	0.67	0.55, 0.81	< 0.001	0.72	0.62, 0.85	< 0.001
2020	0.91	0.80, 1.04	0.154	0.98	0.87, 1.11	0.767	0.94	0.72, 1.21	0.612	0.91	0.73, 1.13	0.375
Province												
Copperbelt	Ref.	–	–	Ref.	–	–	Ref.	–	–	Ref.	–	–
Eastern	2.29	0.99, 5.29	0.052	1.80	0.78, 4.14	0.167	2.35	0.99, 5.59	0.053	1.23	0.53, 2.89	0.628
Luapula	4.87	2.08, 11.4	< 0.001	3.93	1.69, 9.15	0.002	6.30	2.64, 15.1	< 0.001	3.28	1.39, 7.73	0.007
Muchinga	3.09	1.24, 7.68	0.015	2.51	1.02, 6.22	0.046	2.30	0.90, 5.88	0.082	1.31	0.52, 3.30	0.572
Northern	2.54	1.09, 5.94	0.031	2.11	0.91, 4.91	0.084	2.49	1.04, 5.95	0.041	1.51	0.64, 3.57	0.345
North Western	4.16	1.75, 9.92	0.001	3.76	1.58, 8.92	0.003	3.17	1.30, 7.76	0.011	2.29	0.95, 5.52	0.065
Western	1.05	0.47, 2.35	0.901	1.17	0.53, 2.60	0.696	1.81	0.78, 4.17	0.164	1.33	0.59, 3.02	0.489

^a^Rainfall anomalies are lagged and standardized. 3-month lags were used in inpatient deaths models, and 2-month lag in admissions models

To further contextualize the findings, example scenarios were generated to describe the reductions in severe malaria admissions that could be expected from defined increases in malaria service point densities, and the corresponding new CHWs that would be required. Table 4 presents the estimated reductions in severe malaria admissions among all-ages and among children under five that would be expected in each province if all districts had either one service point per 1000, or one service point per 750 population during the final 12 months of the study period.

**Table 4:** Estimated annual reduction in severe malaria admissions among all-ages and among children under five years that could have been achieved with increases in malaria service point density to either 1 per 1,000 population or 1 per 750 population in the final 12 months of the study period (June 2019 to May 2020)*

Province	# Districts with existing CCM scale-up (%)	Mean (min, max) population per malaria service point in a district, June 2019 to May 2020	# Additional CHWs required to reach 1 malaria service point per 1000	# Additional CHWs required to reach 1 malaria service point per 750	All-age severe malaria admissions, June 2019 to May 2020			U5 severe malaria admissions, June 2019 to May 2020		
					Observed (model prediction)	Estimated reduction with 1 malaria service point per 1000	Estimated reduction with 1 malaria service point per 750	Observed (model prediction)	Estimated reduction with 1 malaria service point per 1000	Estimated reduction with 1 malaria service point per 750
Copperbelt	4 (40%)	4903 (601, 9245)	1523	2207	10,510 (11,784)	10.8%	14.7%	3340 (3313)	12.9%	17.5%
Eastern	4 (30%)	3244 (458, 6736)	726	1025	6844 (5960)	8.3%	11.3%	3743 (3291)	10.4%	14.1%
Luapula	1 (8%)	5719 (588, 12,721)	916	1290	14,503 (16,340)	10.5%	14.2%	9112 (12,369)	10.7%	14.3%
Muchinga	3 (33%)	6400 (1173, 11,249)	684	1036	6205 (7074)	12.8%	17.9%	3015 (3499)	15.0%	20.9%
Northern	2 (17%)	6700 (814, 9576)	1228	1736	8875 (9243)	13.0%	17.6%	4480 (4595)	15.2%	20.5%
North Western	7 (64%)	2648 (431, 7976)	561	903	9702 (10,457)	10.1%	14.7%	4174 (4319)	11.5%	16.6%
Western	14 (88%)	1238 (461, 5025)	270	462	2679 (2686)	2.0%	3.3%	1048 (1069)	2.1%	3.6%

^*^Estimated % reductions in severe malaria admissions were produced by comparing the model prediction during June 2019 to May 2020 (using the observed district-month service point density) against model predictions for the two counterfactual scenarios of all districts achieving 1 malaria service point per 1000 population or 1 malaria service point per 750 people

## Discussion

This study presents evidence for the reduction in severe malaria admissions and deaths associated with increasing access to malaria diagnostic and treatment services, primarily through the introduction of community case management of malaria. Using a dose–response approach to analyze routine observational data, a strong relationship was identified between increasing malaria service availability and reduced severe malaria admissions and malaria deaths after accounting for vector control interventions, transmission season and environmental variations. Results suggest an increase of one malaria service point per 1000 is associated with a 19% reduction in severe malaria admissions among U5s and 23% reduction in malaria deaths among U5s. The use of CHWs to deliver CCM has broad support, but evidence of the impact of CCM at large-scale and in routine operational contexts has been limited to date. The current evidence is expected to support continued expansion of CCM, including leveraging of funding from agencies such as the Global Fund against AIDS, Tuberculosis and Malaria to support ongoing CCM implementation costs.

As routine surveillance data on malaria has improved following investments over the past decade, there is growing interest in the use of routine data for evaluating programmatic interventions [20]. While there remain challenges in use and interpretation of routine data [21], routine malaria surveillance data have been used successfully in impact evaluations in Zambia and elsewhere [22, 23]. This analysis leveraged existing routine data to generate the primary exposure and outcome variables. While routine data are imperfect, the key potential biases identified in these data would be expected to bias these findings towards the null effect of no impact of CCM on severe malaria or deaths. Firstly, to account for varying levels of activity or drop-out of CHWs from CCM activities, a CHW was excluded from the total malaria service point count if they did not report any malaria tests for a period of greater than six months. This conservatively may have under-estimated the number of malaria service points operating in any district-month, likely biasing the analysis towards the null hypothesis of no association. Secondly, it is possible that not all malaria-attributable deaths in this population occurred at health facilities; the malaria deaths outcome used in these models does not include any malaria deaths at home and is likely an underestimate of all malaria deaths during the district-month. It was hypothesized that introduction of CCM would either have no impact on malaria deaths occurring at home among those not seeking care, or that CCM would result in increased referral of severely ill individuals to health facilities, potentially increasing the proportion of severe malaria cases and malaria deaths recorded in routine data. Therefore, failing to count malaria deaths beyond the health facilities is expected to result in a potential bias towards the null of no impact of CCM on malaria deaths.

Several assumptions were made in this analysis. The HMIS system does not store zero values for indicators, consequently it is not possible to differentiate between missing indicators for a specific facility-month and a reported zero value. While data cleaning attempted to identify and impute anomalous missing values, it is possible that some missing data was incorrectly considered as a zero value. However, the small number of flagged anomalous values, and long-term investments in strengthening Zambia’s malaria surveillance data suggest that the data are valid for the purposes of this analysis. Vector control data were assembled for this analysis from existing programmatic sources, and used simple assumptions to estimate coverage (e.g. a three-year LLIN life, binary classification of IRS ‘effect’ which persisted for six months following spraying). This analysis did not seek to explicitly estimate the impact of vector control on the outcomes, but simply account for presence and intensity of vector control interventions by district over time. Consequently, there is a risk of bias to these findings only if instances of incomplete or inaccurate vector control data are associated with either CCM (exposure) or severe malaria admissions or deaths (outcome). Due to lack of data, it was not possible to incorporate any other small-scale interventions or community activities which may have influenced healthcare-seeking behaviour, malaria transmission, or broader health status and vulnerability to severe malaria (e.g. anaemia, nutrition).

A central aim of CCM is to improve access to malaria diagnosis and treatment services, by reducing the travel time for individuals to seek care from a qualified care provider. The impact of CCM is therefore partly dependent on appropriate targeting of CCM to the communities furthest from existing health services and most affected by health inequities [24, 25]. The analysis did not consider the distribution of CHWs within a district, only the total service points (CHW or health facilities) operating at any time within the district. A spatially-explicit approach to this analysis could further investigate how CCM impact differs according to remoteness, or identify priority sites for further CCM scale-up.

Models of community health worker service delivery and CCM vary between and even within countries. Zambia delivers CCM through mostly volunteer CHWs. The current findings of CCM impact on severe malaria and malaria deaths may have limited external validity for settings applying a different CCM model. Furthermore, the process for selection of CHWs, extent of training, supervision structures, and other responsibilities may also influence CCM effectiveness across different settings.

## Conclusions

These results show CCM is an effective strategy for preventing severe malaria and deaths in areas such as Zambia where access to malaria diagnosis and treatment remains challenging. These results support the continued investment for scaling-up CCM in similar settings where access to formal health facilities is limited, especially among the most rural and poor communities where risk of dying from malaria is greatest.

## Supplementary Information

**Additional file 1: Figure S1.** Stacked area plot of number of health facilities and community health workers operating by month and province (left) and line plot of total inpatient admissions with confirmed malaria among all ages by month and province (right). Note that y-axes are variable between Provinces.

## Data Availability

Zambia National Malaria Elimination Centre remain the final owners of these data. District-level aggregate data will be available from the corresponding author to researchers who have been granted permission by the NMEC.
