# Supplementary material for: Effectiveness of community case management of malaria on severe malaria and inpatient malaria deaths in Zambia: a dose–response study using routine health information system data
Source: Malar J. 2023 Mar 17;22:96. doi: 10.1186/s12936-023-04525-2 (PMC10022244; doi:10.1186/s12936-023-04525-2)
Supplement: Supplementary file 1 — Additional file 1: Figure S1. Stacked area plot of number of health facilities and community health workers operating by month and province (left) and line plot of total inpatient admissions with confirmed malaria among all ages by month and province (right). Note that y-axes are variable between Provinces. [file 12936_2023_4525_MOESM1_ESM.docx]

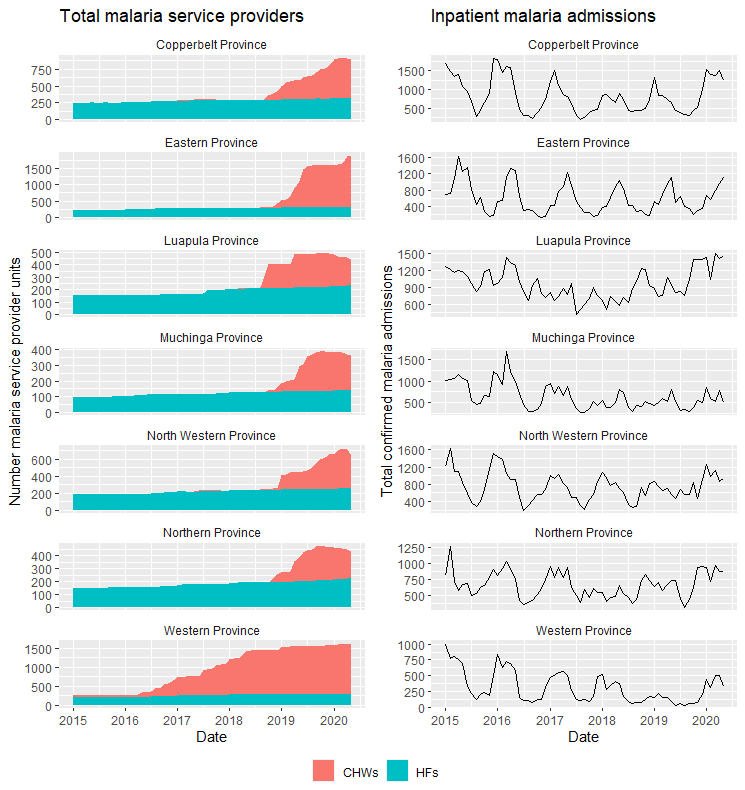
**Supplementary figure 1**  Stacked area plot of number of health facilities and community health workers operating by month and province (left) and line plot of total inpatient admissions with confirmed malaria among all ages by month and province (right). Note that y-axes are variable between Provinces.
